# Experimental evidence on the role of shared protocols as coordination device on clinical best practices

**DOI:** 10.1038/s41598-024-60186-4

**Published:** 2024-04-23

**Authors:** Massimo Finocchiaro Castro, Domenico Lisi, Domenica Romeo

**Affiliations:** 1 Department of Law, Economics and Social Science, Mediterranean University of Reggio Calabria, Reggio Calabria, Italy; 2 Health Econometrics and Data Group, University of York, York, UK; 3 Institute for Corruption Studies, Illinois State University, Normal, USA; 4 Department of Economics and Business, University of Catania, Catania, Italy

**Keywords:** Human behaviour, Health care economics, Health policy

## Abstract

Our experiment assesses the level of coordination on clinical best practice among physicians and investigates whether the release of guidelines helps in supporting coordination. Based on three clinical vignettes using current national guidelines, physicians evaluate the appropriateness of each of the proposed courses of action. Afterwards, physicians are allowed to ask which action corresponds to national guidelines and change their ratings, if desired. On average, slightly more than half of the sample coordinated on appropriateness evaluations. Empirical analysis indicates that several organizational and individual variables influence the level of coordination. Additionally, the release of national guidelines improved both the level of conformity and coordination. Our findings suggest changes in implementation practices to increase the impact of these shared protocols in the health field.

## Introduction

Variation in physicians’ treatment choice is remarkable, and their decision-making is still not fully understood. Previous literature on geographic variations (also known as “small area variations”) has documented that physicians in different areas can, and frequently do, make different treatment choices even for patients with similar profiles (e.g.,^1–5^). Several recent studies provide empirical evidence that many situational factors (such as, defensive medicine, professional norms, technology endowment, availability heuristic) affect physicians’ treatment decisions. Among others, they refer to physicians’ treatment style about prescription drugs (e.g.,^6,7^), the duration of primary care office visits (e.g.,^8,9^), the application of surgical procedures (e.g.,^10,11^).

Overall, this evidence suggests that providers of medical care may not systematically choose the optimal treatment for their patients. Phelps has shown that the welfare loss from an inappropriate medical practice is substantial and increases with the variation (after known patients’ characteristics are considered) in the patterns of use (see^12,13^). This conflict is also underlined in the medical literature in which the need for more coordination on the same clinical practices is regularly advocated (e.g.,^14–16^). Thus, understanding how treatment decisions are made is crucial, also to inform policies addressed to physicians towards the social optimum.

At the same time, the role of information and its diffusion among agents have been largely acknowledged in economics^17^. In the healthcare sector, the problem of incomplete information is pervasive, and the effects can be traced in almost every circumstance^18^. The advance in medical knowledge has markedly expanded the treatment options for many diagnoses. Physicians must confront a large set of treatment technologies in terms of their ability to cure patients, side effects, and costs in a setting where treatment effects may greatly vary according to the unobservable characteristics of the patient. Although desirable, this has increased the uncertainty faced on the selection of treatments for a specific patient, and thus the cognitive effort for physicians’ decision process^10^. Medical practice thus depends on physicians’ beliefs and updating of their knowledge through scientific information and recommendations released by health authorities. However, the public good nature of information in medicine, along with the failure of laws to clearly define property rights on new treatment practices, shape an institutional context with little economic incentive to acquire and disseminate information. In this perspective, the role of government is of paramount importance to ensure a widespread dissemination of information. Phelps provides a deep discussion on the information problems arising in healthcare markets^12^.

Guidelines (also known as shared protocols) are, on the one hand, employed in healthcare with the aim of improving coordination on best practices, and thus patient outcomes and resource saving; on the other hand, they could prevent the adoption of innovative techniques or treatment practices in ever-evolving specialties^19,20^. Guidelines can be classified as soft law instruments that assist in making practitioners’ actions more uniform by identifying recommended courses of action under certain circumstances^21^. While several studies have documented that physicians have heterogeneous beliefs and knowledge (e.g.,^22–24^), the literature on the role of public recommendations in affecting physicians’ behavior is still very limited^25–27^. Physicians agree that guidelines foster coordination on clinical best practices and, in turn, the quality of care^28^. Hence, understanding whether and to what extent the release of guidelines improve coordination of physicians on clinical best practices is a crucial task for healthcare research.

To assess the level of coordination among agents, a recent experimental literature has developed a simple choice mechanism that transforms the elicitation task in a coordination game^29–32^. In this choice framework, players are induced to tacitly coordinate with others in rating actions. Hence, this mechanism enables to investigate the level of coordination among agents, which we employ in our study to assess coordination among physicians in providing patient care.

The purpose of this study is twofold. First, we assess in an artefactual field experiment the level of coordination among physicians in the evaluation of appropriate treatments for some medical conditions. Second, we test whether the release of guidelines on appropriate medical treatments enhances coordination among physicians.

Our behavioral data show that in 51% of appropriateness ratings physicians were able to coordinate. As for determinants of coordination, the empirical analysis suggests that coordination increases when physicians exchange opinions and share positive feedbacks with colleagues. In addition, the presence of a leader in the medical ward facilitates coordination on clinical best practices as recommended by guidelines. Finally, we find that the release of national guidelines significantly improves the level of coordination and conformity to clinical best practices, with relevant implications for healthcare policy.

The novelty of our paper is twofold. To the best of our knowledge, this is the first study assessing the level of coordination among physicians on clinical best practices and the role of guidelines in an experimental setting. The advantage of the experimental approach in this context is that in a controlled setting one can fully attribute the variation in appropriateness ratings to differences in physicians’ beliefs. Second, while other artefactual field and laboratory experiments have involved physicians (e.g.,^33,34^), this is the first artefactual field experiment conducted in the real working environment of physicians (i.e., the hospital). Moreover, in our experiment we employ a significantly large set of hospital physicians (N = 100), as compared to previous experimental health studies.

## Methods

### Experimental design

A mix of hypothetical situations by means of ad hoc vignettes and coordination game design has been used to evaluate physicians’ level of coordination and to study the role of health care guidelines^29,30,32^. Supported by three medical specialists (orthopedist, pediatrician, and oncologist, respectively) not taking part in the experiment, we selected three diagnoses respect of which there should not be huge variation in the evaluations of possible treatments, and then they helped us to properly design the vignettes. The main advantage of the experimental setting is that the characteristics of patients are common knowledge to all physicians, thus the variation in treatment evaluations can be fully attributed to differences in physicians’ beliefs. As a double-check, a general practitioner evaluated the scenarios described in the vignettes (see the Appendix) as realistic and easy understandable to any physicians.

Our artefactual field experiment consists of two treatments: the Coordination Treatment (CT) and the Information Treatment (IT). Before starting the experiment, participants randomly joined one of the two treatments, either (CT) or (IT). Both CT and IT contains two stages. Prior to begin the first stage of each treatment, subjects performed the Holt and Laury’s test^35^ to evaluate their attitude towards risk. Once they have completed the test, the first stage, common to both treatments, starts and participants receive three vignettes, each describing a different patient affected by a specific disease provided with a given diagnosis. The experimental design offers, for each vignette, four actions in response to the disease (one of the four actions reflects the national guidelines suggested for that specific health problem). Participants, then, assess each of the alternative proposed actions on a scale of one to four, based on their perceived degree of appropriateness, where 4 stands for ‘very appropriate’ and 1 for ‘very inappropriate’, being told that, to be awarded the prize, their appropriateness assessments should match the modal assessment obtained in their session. Prior to start the second stage, physicians report their confidence levels about the evaluations made in each vignette on a five-point scale, where 5 stands for most certain^36,37^.

In the second stage of both CT and IT, participants, for each vignette, has the chance to be informed on which of the proposed actions corresponded to national guidelines; if they are not interested, they move to the next vignette. Only the physicians asking for the guidelines’ content receive the information and then start the next vignette. In the IT, participants who are informed on guidelines’ content have the chance to update their assessment of appropriateness. Theoretically speaking, allowing physicians to be aware of guidelines may cause selection-bias in the appropriateness ratings. However, we can quietly exclude such effect because of the very low number of participants not asking for guidelines’ content (3 out of 49 physicians). Also, 2 out of those 3 physicians have already rated the action corresponding to the guidelines as the most appropriate in the first stage of the IT for the three scenarios.

Our experimental design allows us to assess the level of coordination on (and conformity to) clinical best practices among physicians treating the “same” patients, and then to investigate the effects of released guidelines on physicians’ treatment decisions. Evaluating the appropriateness of the courses of action recommended by the national guidelines, as well as the effect of guidelines on the adoption of new treatment practices, are clearly out of the scope of the paper.

Following Krupka and Weber, at the end of the whole experiment, one of the 12 actions is randomly drawn and all choices, within each session, are matched with the modal rating^31^. Those, whose selected evaluation match the modal one, get paid privately. In the CT, the modal evaluations are obtained based on the appropriateness ratings given in the first stage only; differently, in the IT, they are computed using both the evaluations provided in the first stage by the participants not changing their ratings, together and the updated evaluations provided by those participants who have modified their appropriateness ratings. The payment mechanism has been explained in detail to subjects at the start of each session and is described in the instructions.

The experimental sessions have taken place at the two main hospitals of Reggio Calabria, based on an agreement on joint research projects signed with the Mediterranean University of Reggio Calabria. The set of participants counts 100 medical doctors, with different specialties, employed at the hospitals of Reggio Calabria. They have been randomly allocated to treatments: 51 medical doctors (20 women) to the CT and 49 medical doctors (23 women) to the IT. The recruitment has been advertised by means of doctors’ mailing list and by head doctors of all the specialty departments involved, leading to a satisfactory response rate of 49%. The experimental sessions have been conducted in the hospital meeting room and the doctors participated during their coffee-breaks to avoid any interference with the working schedule. To rule out any behavioral spillovers (i.e., any interaction between doctors that have already completed the tasks with those who were about to participate), subjects have accessed the meeting room through one door and, once they completed the tasks, left the room through a different door opening on another area of the hospital. In addition, no more than one session has been run at the same ward to avoid the risk of communication between physicians.

16 sessions have been run with variable number of participants, from a minimum of five to a maximum of eight. Physicians have been not aware of session composition in terms of size and specialty, to rule out any spillover effects on their first-order and second-order beliefs, being the latter crucial to the awarding mechanism. Eight sessions have been run between October and November 2020, whereas the remaining eight between October and November 2021, due to Covid-19 restrictions meanwhile. For, we have tested whether there are any significant differences between the two groups of sessions in terms of physicians’ evaluations. Then Mann–Whitney test fails to reject the null hypothesis of no significant difference (*p* value > 0.1).

In coordination games, players try to guess other players’ behavior^38^ and individual risk attitude may play a role on the outcome of the game, like in a lottery^39^. To assess physicians’ level of risk aversion, we have adopted the well-known test proposed by Holt and Laury^35^ with hypothetical rewards^40^. Results show that 52% of physicians can be classified as risk-averse, 28% as risk-loving, and 20% shown inconsistent behavior. Given that the percentage of risk-lovers is slightly higher than the average level across the experimental literature, we will control for this aspect in the regression analysis.

At the end of Holt and Laury’s test^35^, each subject has taken part into one of the two paper-based treatments. Each treatment has lasted on average 15 min. After completing the experiment, participants have completed a questionnaire on demographic, economic, and job-related questions. Physicians who provided appropriateness evaluation matching the modal answer have earned 10-euro meal ticket exchangeable at the hospital cafeteria. The award is reasonably salient for at least two reasons. First, that cafeteria is the only option available to physicians within the hospital. The closest external to the hospital alternative to the cafeteria would require physicians to walk for 15 min. Moreover, according to hospital regulation, the internal cafeteria charges discounted rates (20% less than standard prices) to hospital’s employees.

Modal answers have been calculated for each session, even in several sessions modal answers overlap. On average, physicians earned €3.70. Although the monetary incentive could be relatively low compared to the average income of the sample, intrinsic motivation should be at work to incentivize their performance. As suggested by Gneezy and Rustichini, the adoption of monetary reward when a specific task has already a motivation, such as joining academic research, may negatively affect individual’s performance^41^. Under certain conditions, the implementation of performance-based reward may crowd out those endogenous incentives that the experimental design attempts at eliciting^42^. All experimental sessions have been conducted according to the relevant guidelines and regulation. Our study received the ethical approval from the Ethics Committee of the Hospitals of Reggio. Also, all participants gave informed consent before the start of the experimental sessions.

### Hypotheses and results

Physicians’ treatment decisions are largely driven by their beliefs and knowledge on uncertain and idiosyncratic-to-patients treatment effects. The acquisition and update of ever-evolving knowledge on treatment options stem from two main sources: physicians’ own experience and evidence-based clinical literature^43^.

Nowadays, the production of clinical guidelines has been considerably spreading^44–46^. Less experienced physicians who cannot rely upon consistent acquired skills are more likely to welcome new information provided by clinical literature and practice guidelines^47^. More experienced physicians, instead, tent to be less willing to adhere to practice guidelines. However, all physicians bear many non-insurable costs incurred for malpractice litigation (such as, time costs and the risk of undermining their reputation) which may lead them to conform to guidelines regardless of their experience level^43,48^. In summary, physicians must bear in mind both clinical literature and practice guidelines while treating their patients, without neglecting their own clinical experience.

From the above discussion on physicians’ behavior, we can make some hypotheses to be tested in the experiment. Although physicians consider their experience in making treatment decisions, when they disagree with their trusted colleagues, their opinions may change. For instance, Gabbay and Le May show that physicians tend to change their opinions while interacting with trusted colleagues and conform to their course of action^49^. This is consistent with the large empirical evidence on heuristics and norms following behavior (e.g.,^6–8,10^). Hence, we expect that physicians tend to coordinate, at least in part, in rating the appropriateness of each of the courses of action proposed as possible solution to a given clinical case. Even though participants to the experiment may not be able to exactly predict how their colleagues will answer to each of the proposed vignettes, they should be able to guess the most likely appropriateness answer and stick with it.

#### Behavioral hypothesis 1

Physicians coordinate in rating the appropriateness of each possible action.

Second, we look more specifically at the coordination on best practices as recommended by guidelines. Even if they are not taken as directives, guidelines generally identify recommended courses of action under certain circumstances^21^. Therefore, we expect that the frequency of assessing the recommended courses of action as ‘very appropriate’ is higher than the average frequency for any other course of action. Additionally, we expect that physicians coordinate in giving the same appropriateness rating to the actions recommended by guidelines more than they do for the other actions.

Behavioral hypothesis 2a: *The average frequency of rating an action corresponding to the guidelines as ’very appropriate’ is higher than the frequency for any other course of action.*

#### Behavioral hypothesis 2b

The average frequency of coordination on actions corresponding to the guidelines is higher than the frequency for any other course of action.

Finally, we investigate whether the release of guidelines leads physicians to switch to the actions recommended by them. Insights from previous literature suggest that physicians’ beliefs are crucial to explaining their treatment decisions, and that their beliefs are affected by new scientific knowledge^12,25–27,50^. So, we expect that physicians could choose to conform to guidelines on what is the best practice to adopt, overruling their previous opinions. This may be due to both following an action shared with colleagues and reducing the risk of being sued for medical malpractice^43,48^. As suggested by Carrier et al., this should also increase coordination of physicians on clinical best practices^28^.

#### Behavioral hypothesis 3a

Once knowing the guidelines, physicians change their appropriateness ranking to the proposed courses of action.

#### Behavioral hypothesis 3b

Once knowing the guidelines, the level of coordination among physicians increases.

### Descriptive analysis and non-parametric tests

Table 1 reports the average frequency of coordination (a subject coordinates when her appropriateness evaluation matches the modal assessment for the specific action considered), the average frequency of coordination on national guidelines, and the average appropriateness evaluation of guidelines across the three vignettes.

**Table 1:** Average frequency of coordination across the experiment.

Vignette	Coordination	Coordination on guidelines	Av. evaluation of guidelines
1—joint effusion on the knee	0.49	0.75	3.69
2—breast fissures	0.52	0.61	3.41
3—cancer of the oral cavity	0.51	0.58	3.46
Overall	0.51	0.65	3.52

#### Result 1

The overall average frequency coordination is 0.51. The non-parametric analysis shows that the differences across vignettes are not significant according to the Friedman test (*p* value < 0.1). Hence, considering all the possible actions, Hypothesis 1 is only partially supported by the experimental evidence.

Then, we look at physicians’ attitude towards national guidelines. As expected, almost all of physicians showed interest in guidelines content (94%). Only six physicians, evenly distributed between treatments, have not asked for guidelines content, having already evaluated in the first stage the action corresponding to guidelines as the most appropriate one in 10 of the 18 evaluations (3 evaluations for each of the six physicians). Table 1 reports that the actions corresponding to guidelines achieved very high average appropriateness ratings (3.52 on average). By comparing the distributions of the statement ‘very appropriate’ (i.e., evaluation = 4) for national guidelines with the distributions of other statements, the Wilcoxon signed-rank test reports significant differences (*p* value < 0.001) in each vignette.

#### Result 2a

Consistently with hypothesis 2a, guidelines’ evaluations have been higher than any other action suggested in the experiment (Wilcoxon signed rank test, *p* value < 0.001).

Moreover, Table 1 shows that the overall average frequency of coordination on national guidelines accounts for 65% of the cases. Thus, the role of national guidelines as a tool to coordinate among physicians cannot be neglected. Additionally, differences in average coordination levels among vignettes are significant (Friedman test, *p* value < 0.05).

#### Result 2b

Consistently with hypothesis 2b, the coordination levels on guidelines are significantly higher than those on all the other actions proposed (Wilcoxon signed rank test, *p* value < 0.001).

Looking closely at the Information treatment, we check whether physicians have changed their first decisions after being provided with national guidelines. We also investigate whether guidelines’ introduction has driven physicians to rate the action corresponding to national guidelines as the most appropriate action (conformity, hereafter), in each vignette.

#### Result 3a

In 21% of the cases, physicians change their previous decisions. Consistently with hypothesis 3a, as shown by Table 2, the average frequency of conformity goes from 0.63 (i.e., prior to the release of guidelines) to 0.74 (i.e., once physicians have the possibility of changing their ratings), and the differences are significant according to Wilcoxon signed rank test (*p* value < 0.001). Hence, national guidelines represent an example of shared protocols among physicians leading to an increase in the level of conformity.

**Table 2:** Average frequency of coordination in the Information Treatment^*^.

Vignette	Ex-ante conformity frequency	Ex-post conformity frequency	Ex-ante coordination frequency	Ex-post coordination frequency
1—joint effusion on the knee	0.68	0.74	0.47	0.48
2—breast fissures	0.64	0.80	0.52	0.58
3—cancer of the oral cavity	0.56	0.68	0.51	0.54
Overall	0.63	0.74	0.50	0.53

*Ex-ante(ex-post) refers to evaluations given by physicians before (after) having the possibility to change ratings.

Table 2 also reports the average physicians’ coordination levels reached before and after the possibility of modifying their own appropriateness assessment for each vignette in the IT.

#### Result 3b

Consistently with hypothesis 3b, on average, physicians increase coordination from 0.50 to 0.53. The increase in coordination is statistically significant (Wilcoxon signed rank test, *p* value < 0.05). Hence, national guidelines have boosted coordination levels among physicians.

To conclude, comparing the appropriateness ratings given in the two stages of the IT, we observe a statistically significant increase in the coordination level (Wilcoxon signed rank test, *p* value < 0.001). Similarly, comparing the coordination levels on national guidelines achieved in the CT with those achieved in the IT, differences are weakly statistically significant (Wilcoxon rank-sum test, *p* value < 0.1). Hence, the effect of shared protocols, as the national guidelines, on the increase of coordination level deserves full consideration.

### Regression analysis

Based on non-parametric results, we investigate which are the determinants of overall coordination and coordination on national guidelines. In Appendix B, Table 1.B reports descriptive statistics of the variables employed in the regression analysis.

Table 3 displays the results of a logit regression. For simplicity, we report the marginal effects of each regressor. The dependent variable in this regression is ‘coordination’, a dummy variable equal to 1 when the physician matches the modal answer and 0 otherwise. Clustered robust standard errors at the individual level have been used to account for data being obtained from multiple observations per physician^51^. We start with the most parsimonious model, and then gradually we add on controls.

**Table 3:** Logit for coordination.

Variables	(1)	(2)	(3)
Confidence	− 0.027	− 0.024	− 0.0205
	(0.0217)	(0.022)	(0.024)
Specialty	0.225***	0.234***	0.234***
	(0.056)	(0.056)	(0.057)
Leader	− 0.0248	0.0127	0.012
	(0.035)	(0.032)	(0.032)
Male		0.0019	0.0014
		(0.0325)	(0.032)
Age		− 0.008***	− 0.008***
		(0.002)	(0.0023)
Years of service		0.0028	0.0028
		(0.003)	(0.0026)
Updating		− 0.034	− 0.033
		(0.029)	(0.029)
Whatsapp		− 0.009	− 0.009
		(0.036)	(0.0367)
Risk seeking		− 0.025	− 0.0251
		(0.034)	(0.034)
Positive influence		0.052*	0.052*
		(0.029)	(0.028)
Negative influence		− 0.069**	− 0.067**
		(0.028)	(0.0284)
Vignette1			− 0.028
			(0.042)
Vignette2			− 0.013
			(0.049)
Constant	0.357	2.322***	2.307***
	(0.310)	(0.839)	(0.846)
Observations	1148	1060	1060

Robust standard errors in parentheses; ***p < 0.01, **p < 0.05, *p < 0.1; Please notice that the number of observations changes from one model to another mainly because of the variable ‘risk seeking’ (due to the exclusion of subjects whose inconsistent choices in the HL questionnaire have prevented them from being classified as either risk-seeker, risk-neutral or risk-averse).

Estimates suggest that the probability of coordinating in each vignette rises roughly by 23% on average when physician’s specialty matches the disease to cure. Physicians’ age decreases the probability of coordinating by 8%. In fact, getting older positively contributes to physicians’ cognitive rigidity^52^, making them more prone to follow their own ideas instead of coordinating with colleagues. On the one hand, receiving positive feedbacks from members of physician’s team promotes shared understanding and contributes to group cohesion increasing physicians’ probability to coordinate^53^. On the other hand, although sharing opinions is essential for coordination^54^, problems could arise when ideas do not match. This could explain why negative influence reduces the likelihood of coordination by approximately 7%. If a physician takes a contrasting colleague’s view into proper consideration, this may lead to divergence of interpretation of the clinical case and, thus, to a decrease in coordination.

Then, we look at coordination achieved on the three choices corresponding to national guidelines only (one for each vignette). Table 4 shows that the longer a physician works at the same hospital, the higher the probability of coordinating on assessing guidelines. One additional year of service leads to roughly 2% increase in the probability of coordination. Working for many years with the same colleagues may create team’s familiarity which boosts communication and coordination skills, improving team performance^55^. Differently, one year increase in age reduces physicians’ likelihood to coordinate by almost 2%. As already discussed, older physicians may be less willing to conform with national guidelines^43,52^. Then, risk seeking turns out to negatively affect coordination on guidelines, decreasing the probability of coordination by 16%. The rationale stems from physicians’ preference to accept higher risk levels when following their own ideas instead of coordinating with others, conforming with common opinion^56,57^. Positive influence displays the same sign but a doubled marginal effect with respect to Table 3, whereas negative influence is not significant anymore. Finally, the presence of a leader in physician’s team increases the probability that he coordinates on national guidelines by 13%. Intuitively, when a team is led by a leader, the exchange of ideas and thus of positive feedbacks among colleagues are stimulated which, in turn, may boost coordination.

**Table 4:** Logit model—Dependent variable: Coordination on guidelines.

Variables	(1)	(2)	(3)
Confidence	0.061*	0.075**	0.056
	(0.036)	(0.036)	(0.039)
Specialty	0.31**	0.357***	0.398***
	(0.119)	(0.113)	(0.121)
Leader	− 0.025	0.13*	0.134*
	(0.07)	(0.071)	(0.072)
Male		− 0.116	− 0.1037
		(0.07)	(0.071)
Age		− 0.021***	− 0.022***
		(0.0043)	(0.0045)
Years of service		0.014***	0.015***
		(0.003)	(0.004)
Updating		− 0.006	− 0.012
		(0.0549)	(0.055)
Whatsapp		0.020	0.0094
		(0.071)	(0.072)
Risk seeking		− 0.163**	− 0.167**
		(0.0823)	(0.083)
Positive influence		0.101*	0.107*
		(0.055)	(0.056)
Negative influence		− 0.0255	− 0.031
		(0.048)	(0.050)
Vignette1			0.180**
			(0.089)
Vignette2			− 0.052
			(0.0826503)
Constant	− 0.495	2.543	2.893
	(0.619)	(1.691)	(1.778)
Observations	287	265	265

Robust standard errors in parentheses; ***p < 0.01, **p < 0.05, *p < 0.1.

Finally, we focus on the IT to assess whether the release of guidelines has increased coordination on clinical best practices. Specifically, we combine data on the coordination level of the 49 physicians in the first stage with the coordination level achieved in the second stage, after being exposed to national guidelines. Results of the logit regressions are reported in Table 5, in which the dummy variable ‘guidelines’ is equal to 1 for the second stage observations and 0 otherwise.

**Table 5:** Logit model—Dependent variable: Coordination.

Variables	(1)	(2)	(3)
Confidence	− 0.085***	− 0.067**	− 0.065**
	(0.103)	(0.117)	(0.117)
Specialty	0.232***	0.215***	0.196***
	(0.265)	(0.276)	(0.276)
Leader	0.027	0.047	0.045
	(0.142)	(0.149)	(0.148)
Male		− 0.054	− 0.056
		(0.140)	(0.138)
Age		− 0.006**	− 0.006**
		(0.0100)	(0.0103)
Years of service		0.004	0.004
		(0.0119)	(0.0120)
Updating		− 0.021	− 0.021
		(0.157)	(0.160)
Whatsapp		0.008	0.009
		(0.151)	(0.153)
Riskseeking		0.033	0.033
		(0.167)	(0.166)
Positive influence		− 0.015	− 0.017
		(0.152)	(0.152)
Negative influence		− 0.02	− 0.02
		(0.118)	(0.119)
Vignette1			− 0.04
			(0.232)
Vignette2			0.03
			(0.261)
Guidelines	0.036**	0.036*	0.036*
	(0.068)	(0.075)	(0.0749)
Constant	1.200***	2.637***	2.579***
	(0.382)	(0.844)	(0.829)
Observations	1136	1048	1048

Robust standard errors in parentheses; ***p < 0.01, **p < 0.05, *p < 0.1.

Estimates from Table 5 confirms the positive role played by the release of guidelines. Providing physicians with guidelines content, though mildly, increases the coordination level among physicians, consistently with hypothesis 3b and the non-parametric analysis. Therefore, national guidelines implementation can help to reduce treatment variation and increase the level of coordination on clinical best practices.

## Discussion

Our artefactual field experiment has assessed the level of coordination among physicians and adherence to national guidelines, as well as their role to enhance coordination on clinical best practice. The average level of coordination reached in the experiment amounted to 51%. The empirical analysis pointed out that coordination increases when physicians exchange opinions and share positive feedbacks with members of their own team. Moreover, the presence of a leader in a medical ward turned out to favor coordination on clinical guidelines. As for the informative role of guidelines, our results showed that their release significantly improves both the level of coordination and conformity to best practices.

Our study relates to different strands of literature. First, it integrates the still limited literature on the effects of information and public recommendations on physicians’ decision making. The key insight from this literature is that physicians’ beliefs are crucial to explaining their treatment choices, and that their beliefs are affected by a widespread diffusion of new scientific knowledge^25–27^. We contribute to this stream of research by adding experimental evidence on the role of guidelines as an informative tool to increase coordination among physicians. Second, our study relates to the recent experimental literature employing coordination games to assess coordination among individuals in their consideration of appropriate behaviors^29–32,58,59^. Most of these papers employ this approach to measure the extent of individuals’ beliefs on social norms. As suggested by Fallucchi and Nosenzo^59^, however, when there are clear expectations on what constitutes appropriate behavior the Krupka and Weber’s method^31^ can be employed to study individuals’ beliefs about appropriateness ratings on different courses of action. In the health sector, there are indeed clear shared expectations among physicians on the appropriateness of possible treatments (at least, there are for the three diagnoses employed in our experiment), which are represented by guidelines. Therefore, we apply for the first time this experimental approach using real hospital physicians to assess their level of coordination on clinical best practices. Finally, our study provides support to the use of vignettes as a tool to find out what people think about several topics^60,61^. Clinical vignettes have also been used to assess specific features of physicians^62,63^ such as treatment choices^64^, confidence levels^65^, clinical experience^66^.

### Policy implications

Our study raises key implications for healthcare policy. Not only should hospitals implement guidelines dissemination program but also governments should involve a higher number of physicians in the clinical recommendations designing process. In fact, physicians strive for being involved in the process of adoption of innovations, including new protocols and guidelines. Making physicians part of the research activities through their clinical experience not only could help develop more flexible, comprehensive, and shared guidelines but also could really induce physicians to follow them in practice.

An interesting insight comes from the remarkably high physicians’ willingness to know guidelines (94% of the sample), mostly encouraged by the guidelines’ ease of accessibility in our experimental design. In this respect, introducing a newsletter program and providing an alternative learning option to the standard education courses could be a solution^67^. More than other policy interventions, newsletter would have the features of accessibility and searchability required by physicians.

### Limitations and avenues for further research

Although our experimental evidence contributes to the literature and entails important policy implications (see below) on a relevant topic, in this section we discuss some limitations and avenues for further research. The first limitation is about what our study captures in terms of physicians’ behavior. In this paper we aimed at measuring the extent of coordination among physicians’ beliefs and whether the release of guidelines improves coordination on clinical best practices. The coordination game *a là* Krupka and Weber^31^ is an approach largely employed in the literature to measure the extent of coordination among agents’ beliefs; however, nothing can be said about what this coordination on beliefs comes from. Specifically, in the context of our experiment, coordination among physicians might come from either a non-incentivized convergence of individual assessments on the most appropriate courses of action, or from an incentivized behavior of predicting what other physicians assess as the most appropriate courses of action. While it is not easy to disentangle the two sources of coordination in our experiment, a promising route might be to run an additional experiment including the physicians’ assessments on the appropriateness of the proposed courses of actions without coordination, as in Burks and Krupka^29^.

Another limitation might be given by the extent of the financial incentives for coordination employed in our experiment. The literature is not unanimous on what would be an appropriate extent of the incentive, especially when the behavior investigated in the experiment has its own intrinsic motivation, as it is the case in medical research. For instance, Gneezy and Rustichini^41^ point out that introducing monetary rewards contingent on performance may put down the intrinsic motivation for the behavior the experimenter wants to elicit. In our experiment, the financial incentive employed could be retained relatively low given the average income of real physicians participating in the experiments. While their intrinsic motivation should be already enough to incentivize their behavior, we cannot rule out that the extent of the monetary reward was indeed too low to effectively elicit their behavior. Though our agreement with the Mediterranean University of Reggio Calabria, based on the project “Experiments in Health Economics” makes us confident that elicited behaviors are authentic, further research should investigate the role of the extent of the financial incentives for coordination in the healthcare context.

Finally, a crucial avenue for future research on the role of clinical guidelines in healthcare is about their impact on the adoption of new techniques and treatment practices. While this issue is not considered in this study, the experimental setting employed in the growing experimental health literature might represent a promising approach for further investigation.

### Supplementary Information

Supplementary Information.

## Data Availability

All datasets are available from M.F.C. (massimo.finocchiaro@unirc.it) upon request.
