# Supplementary material for: Experimental evidence on the role of shared protocols as coordination device on clinical best practices
Source: Sci Rep. 2024 Apr 23;14:9363. doi: 10.1038/s41598-024-60186-4 (PMC11039456; doi:10.1038/s41598-024-60186-4)
Supplement: Supplementary file 1 — Supplementary Information. [file 41598_2024_60186_MOESM1_ESM.docx]

**Appendix A**

**Experimental instructions**

Welcome to this experiment.

In this experiment you will be asked to perform some tasks and you will receive a payoff related to it. During the experiment, we request that you remain silent and do not attempt to communicate with other participants. Participants who do not follow this request may be asked to leave without receiving payment. If you have any questions, please raise your hand and one of us will come to you.

At the end of the experiment, you may win a monetary prize, based on your choices and the choices of others in the task described below.

There will be one task for all participants to perform. You will not receive feedback on the outcome of the task, and you will not be paid until the end of the experiment.

**Lottery questionnaire (taken from Holt and Laury 2002)**

Before starting the experiment, please fill the following questionnaire in all its parts. Your answers will not affect your future earnings.

Choose one lottery from the two lotteries proposed.

| **Lottery A** | **Lottery B** | **Your choice** |
| --- | --- | --- |
| 2€ with probability 1/10  1,60€ with probability 9/10 | 3,85€ with probability 1/10  0,10€ with probability 9/10 |  |
| 2€ with probability 2/10  1,60€with probability 8/10 | 3,85€with probability 2/10  0,10€ with probability 8/10 |  |
| 2e with probability 3/10  1,60€ with probability 7/10 | 3,85€ with probability 3/10  0,10€ with probability 7/10 |  |
| 2€ with probability 4/10  1,60€ with probability 6/10 | 3,85€ with probability 4/10  0,10€ with probability 6/10 |  |
| 2€ with probability 5/10  1,60€ with probability 5/10 | 3,85€ with probability 5/10  0,10€ with probability 5/10 |  |
| 2€ with probability 6/10  1,60€ with probability 4/10 | 3,85€ with probability 6/10  0,10€ with probability 4/10 |  |
| 2€ with probability 7/10  1,60€ with probability 3/10 | 3,85€ with probability 7/10  0,10€ with probability 3/10 |  |
| 2€ with probability 8/10  1,60€ with probability 2/10 | 3,85€ with probability 8/10  0,10€ with probability 2/10 |  |
| 2€ with probability 9/10  1,60€ with probability 1/10 | 3,85€ with probability 9/10  0,10€ with probability 1/10 |  |
| 2€ with probability 10/10  1,60€ with probability 0/10 | 3,85€ with probability 10/10  0,10€ with probability 0/10 |  |

Once you have completed the questionnaire, the experiment will start.

**Task one**

You will receive a description of three scenarios. This description corresponds to situations in which one person, “Doctor House,” must decide how to act. You will be given a description of various possible actions Doctor House can choose to take in response to each scenario.

After you have read the description of the scenario, you will be asked to evaluate each of the various possible actions Doctor House can choose to take for each scenario. You must indicate, for each of the possible actions, whether taking that action would be “appropriate” or “inappropriate” on a scale of 1 to 4, where 1 means very inappropriate and 4 means very appropriate. We consider an action to be appropriate when you think is the “correct” thing to do in each specific scenario.

In each of your responses, we would like you to answer as truthfully as possible, based on your opinions of what constitutes appropriate or inappropriate action. To give you an idea of how the experiment will proceed, we will go through an example and show you how you will indicate your responses.

**Example**

Doctor House is treating a patient who has been admitted in the hospital rehab block due to a compound fracture of his shoulder. Doctor House can choose 4 possible actions to take: referring the patient to a fifteen-minute magneto- therapy per day; referring the patient to a forty-minute magneto-therapy per day, referring the patient to a three-hour magneto-therapy per day; referring the patient to a seven-hour magneto-therapy per night.

The table below presents the list of the possible actions Doctor House can choose. For each of the actions, you would be asked to indicate whether you believe choosing that action is very inappropriate, somehow inappropriate, somehow appropriate, or very appropriate. To indicate your response, you would put a cross in the row corresponding to your belief about the degree of appropriateness.

|  | 15-minute | 40-minute | 3-hour | 7-hour |
| --- | --- | --- | --- | --- |
|  | magneto- | magneto- | magneto- | magneto- |
|  | therapy | therapy | therapy | therapy |
|  | per day | per day | per day | per night |
| 1 Very inappropriate |  |  |  |  |
| 2 Somehow inappropriate |  |  |  |  |
| 3 Somehow appropriate |  |  |  |  |
| 4 Very appropriate |  |  |  |  |

For example, suppose you thought that referring the patient to a three-hour magneto-therapy per day was very inappropriate, referring the patient to a fifteen-minute magneto-therapy per day was somehow inappropriate, referring the patient to a three-hour magneto-therapy per day was somehow appropriate and referring the patient to a seven-hour magneto-therapy per night was very appropriate, then you would indicate your responses as follows:

|  | 15-minute | 40-minute | 3-hour | 7-hour |
| --- | --- | --- | --- | --- |
|  | magneto- | magneto- | magneto- | magneto- |
|  | therapy | therapy | therapy | therapy |
|  | per day | per day | per day | per night |
| 1 Very inappropriate |  |  | X |  |
| 2 Somehow inappropriate | X |  |  |  |
| 3 Somehow appropriate |  | X |  |  |
| 4 Very appropriate |  |  |  | X |

Please note that you may give the same level of appropriateness to more than one action within the same vignette.

After completing the task, you will be asked to indicate how much confident do you feel about your choices on a five-point scale, where 5 stands for a great deal of confidence.

If you have any questions about this example or about how to indicate your responses, please raise your hand now and we will assist you privately.

You will next be given the description of three situations where Doctor House faces various possible actions. You will be given a paper with the description of the scenarios and a pen to write down your answer. After you read the description, you must consider the possible actions and indicate on the paper you receive how appropriate these are in a table like the one shown above in the example.

Once you have completed the task, an experimenter will come to collect your paper.

**Payment procedure**

Once all the answers have been collected, a computer will randomly select one scenario and for that scenario one specific action. Your evaluation of this action will be compared with the response selected by the other participants. If your evaluation coincides with the most frequently chosen option, you will receive a 10-euro meal voucher to be used at the cafeteria for this task, otherwise you will receive zero. For instance, imagine the example above was the actual scenario and the possible action “Fifteen-minute magneto-therapy” was selected by the computer. If your evaluation had been “somehow inappropriate” then your task earnings would be 10 euros if that was the response selected by most of participants into your session and zero otherwise.

While the experimenters are calculating your total payoff, we ask you to complete a short, anonymous questionnaire. Please leave the questionnaire on your desk once you have completed it.

**Scenarios**

Now we present three different scenarios similar to the previous example. For each scenario, we propose four actions that Doctor House can take. For each of the actions, we ask you to indicate whether you believe choosing that action is very inappropriate, somehow inappropriate, somehow appropriate, or very appropriate. Recall that by appropriate we mean action that you think is the “correct” thing to do in each specific situation.

**First scenario**

Doctor House deals with a 26-year-old patient who sustained a tear to the anterior cruciate ligament together with a meniscus injury. Doctor House has four different options: performing joint aspiration (arthrocentesis), prescribing cryotherapy and magneto-therapy applications, suggesting surgery treatment, recommending ice and rest. Here is the table reporting all the four available actions. For each of the actions you must indicate whether you believe choosing that action is very inappropriate, somehow inappropriate, somehow appropriate, or very appropriate. Recall that by appropriate we mean action that you think is the “correct” thing to do in each specific situation. To indicate your response, you must put a cross in the row corresponding to your belief about the degree of appropriateness for each of the actions which Doctor House can take. Remember that at the end of the experiment your evaluations will be compared with the most common answers provided today. You will earn monetary prize by matching the most frequently chosen option.

|  | Prescribing magneto  -therapy and cryotherapy | Recommending ice and rest | Surgery treatment | Performing  joint aspiration |
| --- | --- | --- | --- | --- |
| 1 Very inappropriate |  |  |  |  |
| 2 Somehow inappropriate |  |  |  |  |
| 3 Somehow appropriate |  |  |  |  |
| 4 Very appropriate |  |  |  |  |

How much confident do you feel about your choice of optimum treatment? (on a scale 1 to five where 1 stands for no certainty and 5 for a great deal of confidence)

Do you want to know national guidelines content?

- Yes
- No

Do you want to change your previous answers?

- Yes
- No

**Second scenario**

Doctor House deals with a 28-year-old woman who suffers from breast fissures.

Doctor House has four different options: suggesting discontinuing nursing, suggesting discontinuing nursing and in the meanwhile prescribing protective creams, recommending correcting errors in latch, suggesting using pump. Here is the table reporting all the four available actions. For each of the actions you must indicate whether you believe choosing that action is very inappropriate, somehow inappropriate, somehow appropriate, or very appropriate. Recall that by appropriate we mean action that you think is the “correct” thing to do in each specific situation. To indicate your response, you must put a cross in the row corresponding to your belief about the degree of appropriateness for each of the actions which Doctor House can take. Remember that at the end of the experiment your evaluations will be compared with the most common answers provided today. You will earn monetary prize by matching the most frequently chosen option.

|  | Suggesting | Suggesting | Recommending | Suggesting |
| --- | --- | --- | --- | --- |
|  | discontinuing | discontinuing | correcting | using |
|  | nursing | nursing and | errors | pump |
|  |  | prescribing | in latch |  |
|  |  | protective |  |  |
|  |  | creams |  |  |
| 1 Very inappropriate |  |  |  |  |
| 2 Somehow inappropriate |  |  |  |  |
| 3 Somehow appropriate |  |  |  |  |
| 4 Very appropriate |  |  |  |  |

How much confident do you feel about your choice of optimum treatment? (on a scale 1 to five where 1 stands for no certainty and 5 for a great deal of confidence)

Do you want to know national guidelines content?

- Yes
- No

Do you want to change your previous answers?

- Yes
- No

**Third scenario**

Doctor House deals with a 54-year-old patient who has a cancer to the oral cavity

in the retromolar region which involves the pterygoid muscle. Doctor House has four alternate options: treating it with surgery and radiotherapy, treating it with chemotherapy, treating it with radiotherapy, treating it with surgery. Here is the table reporting all the four available actions. For each of the actions you must indicate whether you believe choosing that action is very inappropriate, somehow inappropriate, somehow appropriate, or very appropriate. Recall that by appropriate we mean action that you think is the “correct” thing to do in each specific situation. To indicate your response, you must put a cross in the row corresponding to your belief about the degree of appropriateness for each of the actions which Doctor House can take. Remember that at the end of the experiment your evaluations will be compared with the most common answers provided today. You will earn monetary prize by matching the most frequently chosen option.

|  | Treating it | Treating it | Treating it | Treating it |
| --- | --- | --- | --- | --- |
|  | with | with | with | with |
|  | surgery and | chemotherapy | radiotherapy | surgery |
|  | radiotherapy |  |  |  |
| 1 Very inappropriate |  |  |  |  |
| 2 Somehow inappropriate |  |  |  |  |
| 3 Somehow appropriate |  |  |  |  |
| 4 Very appropriate |  |  |  |  |

How much confident do you feel about your choice of optimum treatment? (on a scale 1 to five where 1 stands for no certainty and 5 for a great deal of confidence)

Do you want to know national guidelines content?

- Yes
- No

Do you want to change your previous answers?

- Yes
- No

**Questionnaire**

**Participant ID . . . . . .**

**Demographic Questionnaire**

The following questions ask for some information about you. Please answer each question by placing a mark where appropriate or by writing a brief response.

If you have a question, please raise your hand and we will come to assist you.

1. **How old are you?** …..
2. **What is your gender?**

- Male
- Female

1. **What best describes your race or ethnicity (please select all that apply)?**

- White
- Black/African/America
- Asian or Pacific Islander
- Hispanic
- Multiracial
- Other

1. **What hospital/clinic do you work for?**..................................................
2. **How long have you been with that hospital/clinic?**.......
3. **In general, how satisfied are you with the hospital/clinic you work in?**

- Very dissatisfied
- Somehow dissatisfied
- Somehow satisfied
- Very satisfied

1. **Which kind of medical specialty do you belong to?**.....................................
2. **How many doctors work in your department?...................**
3. **How often do you ask for colleagues’ advice to take decisions?**

- Never
- Seldom
- Often
- Very often

1. **How much does your colleague’s opinion affect you if he agrees with you?**

- Not at all
- Slightly
- Somehow
- Very much

1. **How much does your colleague’s opinion affect you if he does not agree with you?**

- Not at all
- Slightly
- Somehow
- Very much

1. **How often decisions about a patient are taken because of a team’s valuation?**

- Never
- Seldom
- Often
- Very often

1. **How often do you share stuff with other colleagues (e.g., re- sults of diagnostic tests, outcomes of surgical procedures)?**

- Never
- Seldom
- Often
- Very often

1. **When you work in team**

- a: Any decision taken is the result of a shared opinion
- b: There is a member whose opinion weights the most

1. If you have answered a to question 13, please skip to question 16. **What is the role of the team member whose opinion weights the most?**..........................................
2. **Do you have a whatsapp group with your colleagues where you discuss decisions to take?**

- Yes
- No

1. **How often do you take part in training courses?**

- Never
- Seldom
- Often
- Very often

1. **How often do you take part in brainstorming sessions?**

- Never
- Seldom
- Often
- Very often

1. **How often do you consult scientific journals?**

- Never
- Seldom
- Often
- Very often

1. **Do you carry out and public scientific research?**

- Yes
- No

1. **How important are national guidelines in your decisions?**

- Very unimportant
- Somehow unimportant
- Somehow important
- Very important

1. **Does your firm adopt internal guidelines?**

- Yes
- No

1. If you have answered no to question 22, please skip to question 24. **How important are internal guidelines in your decisions?**

- Very unimportant
- Somehow unimportant
- Somehow important
- Very important

1. **Do you think that your colleagues follow guidelines while taking decisions**

- Never
- Seldom
- Often
- Very often

1. **What is your average opinion of your colleagues?**

- Bad
- Mediocre
- Somehow good
- Very good

1. **What is your average opinion of your colleagues with respect to yourself?**

- Bad
- Mediocre
- Somehow good
- Very good

1. **Do you have an insurance?**

- Yes
- No

1. **How do you judge your department in terms of coordination between colleagues?**

- Bad
- Mediocre
- Somehow good
- Very good

Appendix B

**Table 1.B:** Descriptive statistics

| **Variables** | **Description** | **Mean** | **St.Dev.** | **Min** | **Max** |
| --- | --- | --- | --- | --- | --- |
| Male | Dummy for gender | 0.57 | 0.49 | 0 | 1 |
| Age | Age | 48.82 | 11.28 | 24 | 69 |
| Years of service | Years of employment | 12.49 | 11 | 1 | 43 |
| Confidence | Individual perceived correctness on a five-point scale | 3.71 | 0.79 | 1 | 5 |
| Specialty | Dummy for specialization in the specialty of the vignette | 0.12 | 0.32 | 0 | 1 |
| Updating | Frequency of consulting scientific journals on a f.p.s* | 3.36 | 0.61 | 1 | 4 |
| Negative influence | Influence of colleagues’ divergent view on a f.p.s* | 2.58 | 0.67 | 1 | 4 |
| Positive influence | Influence of colleagues’ concordant view on a f.p.s* | 2.85 | 0.61 | 1 | 4 |
| Leader | Dummy for the presence of a leader in the team | 0.30 | 0.45 | 0 | 1 |
| Whatsapp | Dummy for joining a whatsapp group with ward colleagues | 0.60 | 0.49 | 0 | 1 |
| Risk seeking | Dummy for risk seeking | 0.24 | 0.43 | 0 | 1 |
| Guidelines | Dummy for physicians being exposed to guidelines | 0.5 | 0.5 | 0 | 1 |
| Coordination | Dummy for coordination | 0.508 | 0.50 | 0 | 1 |
| Conformity with guidelines | Dummy for compliance with guidelines | 0.65 | 0.48 | 0 | 1 |
| Vignette1 | Dummy for vignette 1 | 0.33 | 0.47 | 0 | 1 |
| Vignette2 | Dummy for vignette 2 | 0.33 | 0.47 | 0 | 1 |
| Vignette3 | Dummy for vignette 3 | 0.33 | 0.47 | 0 | 1 |

*f.p.s.= Four-point scale

These variables have been obtained from physicians’ answers to the questionnaire submitted at the end of the experiment (see, Appendix). Dummy variables for each vignette are introduced to control for specific effect. *Confidence* is a categorical variable which indicates how physicians state the correctness of their answers to the specific vignette, answering to the question ‘How much certainty do you feel about your choice of optimum treatment? (on a scale 1 to five where 1 stands for no certainty and 5 for a great deal of certainty)’; *Specialty* indicates the physician’ belonging medical department, and specifically it assumes the value 1 when physician’s training regards the field of the vignette (e.g. for an orthopedist, specialty takes the value one for the four actions proposed in the first vignette ‘joint effusion on the knee’ and 0 for the remaining 8 actions); *Updating* is a categorical variable referred to physicians’ frequency of consulting scientific journals on a four-point scale, where higher values imply greater frequency; *Positive(Negative) influence* is a categorical variable which indicates whether and to which extent a colleague’s similar (divergent) view affects physician’s decision on a four-point scale (where higher values stand for greater importance given to the others’ opinion), and is derived from providers’ answers to the question ‘How much does your colleague’s opinion affect you if he agrees with you? (How much does your colleague’s opinion affect you if he does not agree with you?)’; *Leader* is equal to 1 if subjects report that when they work in team there is a member whose opinion weights the most (e.g. head of the department or first operator); *Whatsapp* is a dummy variable referred to whether physicians have a whatsapp group with their colleagues to discuss patients’ treatments; *Risk seeking* is a dummy variable which takes the value 1 for risk taking subjects according to providers’ answers to the HL questionnaire.
